# Improved Diagnostic Accuracy of Bone Metastasis Detection by Water-HAP Associated to Non-Contrast CT

**DOI:** 10.3390/diagnostics10100853

**Published:** 2020-10-20

**Authors:** Yoshinobu Ishiwata, Yojiro Hieda, Soichiro Kaki, Shinjiro Aso, Keiichi Horie, Yusuke Kobayashi, Motoki Nakamura, Kazuhiko Yamada, Tsuneo Yamashiro, Daisuke Utsunomiya

**Affiliations:** 1 Department of Radiology, Yokohama City University Hospital, 3–9 Fukuura, Kanazawa-ward, Yokohama City 2360004, Japan; d25037@gmail.com (S.A.); kei1horie@gmail.com (K.H.); clatsune@yahoo.co.jp (T.Y.); d_utsuno@yokohama-cu.ac.jp (D.U.); 2 Department of Radiology, Odawara Municipal Hospital, 46 Kuno, Odawara City 2508558, Japan; yojiro19@gmail.com (Y.H.); bunkyo93@gmail.com (S.K.); ea3kymd@gmail.com (K.Y.); 3 Department of Radiology, Yokohama City University Medical Center, 4–57 Urafune, Minami-ward, Yokohama City 2320024, Japan; intangibedj2@gmail.com (Y.K.); taremaruo@gmail.com (M.N.)

**Keywords:** neoplasm metastasis, bone marrow, bone neoplasms, cone-beam computed tomography, dual-energy scanned projection radiography

## Abstract

We examined whether water-hydroxyapatite (HAP) images improve the diagnostic accuracy of bone metastasis compared with non-contrast CT alone. We retrospectively evaluated dual-energy computed tomography (DECT) images of 83 cancer patients (bone metastasis, 31; without bone metastasis, 52) from May 2018 to June 2019. Initially, two evaluators examined for bone metastasis on conventional CT images. In the second session, both CT and CT images plus water-HAP images on DECT. The confidence of bone metastasis was scored from 1 (benign) to 5 (malignant). The sensitivity, specificity, positive predictive values, and negative predictive values for both modalities were calculated based on true positive and negative findings. The intra-observer area under curve (AUC) for detecting bone metastasis was compared by receiver operating characteristic analysis. Kappa coefficient calculated the inter-observer agreement. In conventional CT images, sensitivity, specificity, positive predictive value, and negative predictive value of raters 1 and 2 for the identification of bone metastases were 0.742 and 0.710, 0.981 and 0.981, 0.958 and 0.957, and 0.864 and 0.850, respectively. In water-HAP, they were 1.00 and 1.00, 0.981 and 1.00, 0.969 and 1.00, and 1.00 and 1.00, respectively. In CT, AUCs were 0.861 and 0.845 in each observer. On water-HAP images, AUCs were 0.990 and 1.00. Kappa coefficient was 0.964 for CT and 0.976 for water-HAP images. The combination of CT and water-HAP images significantly increased diagnostic accuracy for detecting bone metastasis. Water-HAP images on DECT may enable accurate initial staging, reduced radiation exposure, and cost.

## 1. Introduction

Accurate cancer staging is essential for treatment planning and prognosis. The bone is among the most common sites of metastases, and the identification is clinically important. Bone metastases are classified into four types: sclerotic, osteolytic, mixed, and bone marrow metastases, which is the initial stage of bone metastasis [1,2,3]. Although computed tomography (CT) is generally used to screen for the metastases in the whole body, the detection is challenging because invisible bone metastases on CT images (mainly bone marrow metastases) account for approximately 30% of all bone metastases [3,4,5]. Bone scintigraphy is the current gold standard diagnostic method for carcinomas at a high risk of bone metastasis at the expense of cost and radiation exposure [6]. However, the specificity of the bone scintigraphy is low for the differentiation of benign and malignant uptake, and it is not performed for all cancer patients with a relatively lower risk for bone metastasis, leading to a delay in the detection of bone metastasis. Even in such patients, whole-body CT is usually performed for the evaluation of the metastatic lesions. Therefore, bone metastasis detection by CT may be desirable in clinical practice.

Water-Hydroxyapatite (HAP) images, which provide information on the water density in the bone, can be acquired by dual-energy (DE) CT using two types of X-rays with different tube voltages [7,8]. On the conventional CT, bone metastasis is often difficult to identify because the metastatic lesion is masked by the bone’s high density. In contrast, magnetic resonance imaging (MRI) is considered superior in detecting bone metastases [9] by the process of visualisation of “water molecules” of the metastasis. We hypothesised that water-HAP images can extract the water-rich component in the bone metastasis like MRI and help detect bone metastases with higher sensitivity. This study was performed to test the hypothesis that adding water-HAP images would improve the diagnostic accuracy for bone metastasis of non-contrast CT.

## 2. Materials and Methods

### 2.1. Patient Population

This single-centre, non-interventional, retrospective study was approved by the institutional review board (approval ID: 2020-5; 12 May 2020), and the study was performed as per the principles of the Declaration of Helsinki. Written informed consent was waived due to the retrospective nature of this study.

In this study, we reviewed 191 consecutive patients with prostate cancer alone, as they were followed for a long time with a clear clinical course in the hospital, who underwent non-contrast CT from May 2018 to April 2019. All recruited patients had non-contrast CT for cancer staging or restaging. Inclusion criteria were that DECT was performed, bone scintigraphy should be performed within one month before or after DECT, and the progress can be tracked by electronic medical records for at least 1 year. Exclusion criteria were those who have lost raw data of DECT for image reconstruction. Clinical information was obtained from electronic medical records regarding age, gender, pathological findings of primary cancer, clinical diagnosis, and CT and bone scintigraphy date. The final diagnosis of bone metastases was performed by the subsequent clinical course or positive results of radiological studies, i.e., follow-bone scintigraphy.

### 2.2. Data Acquisition

DECT images were collected by 256-row multidetector CT (Revolution CT, GE Healthcare, Waukesha, USA) with one X-ray tube and one detector with fast switching between two different tube voltages (80 kVp and 140 kVp) at 0.25 ms/view/rotation (fast kVp switching). No contrast agent was used in all cases. CT imaging conditions were as follows: scan type, helical and beam; rotation time, 0.5 s; configuration, 80 mm; helical pitch, 0.992:1; current, 200–485 mA; peak voltage, 80–140 kVp at first kVp switching; kernel, standard; total scan time, 3.0–4.0 s. The mean volume CT dose index (CTDI vol) was 12.4 ± 3.2 mGy/cm. The mean volume CT dose index (CTDI vol) and the mean dose-length product were 12.4 ± 3.2 mGy and 977.5 ± 274.6 mGy/cm, respectively. A standard DECT workstation (GSI Viewer, AW Server 2.02, GE Healthcare, Buc, FRANCE) was used to split the raw data into 80 kV; 140 kV projection data sets to obtain highly accurate projection data by correcting for water and iodine beam hardening artefacts. This correction made it possible to express the pixel values of water and iodine in terms of density values and to create images of various combinations of material densities based on the two material decomposition algorithms. In this study, the combination of water and hydroxyapatite was used to obtain images in which hydroxyapatite, a basic component of bone, was suppressed, and water concentration was enhanced.

Moreover, we used virtual monochromatic spectral (VMS) images of 70 keV as conventional CT because they are equivalent to the Hounsfield unit (HU) of conventional single energy CT [10].

### 2.3. Data Analysis

In image interpretation, the default window level settings for the conventional CT images were in the vertebral window (width, 2000 HU; level, 200 HU). The default window width and level settings of the water-HAP images displayed in colour were 300 mg/cm^3^ and 1020 mg/cm^3^. In the first session, two independent evaluators (radiologists with 6 and 9 years of experience) had a bone metastasis reliability score using the following five-point scale: 1—normal lesion, 2—potentially malignant lesion, 3—probably malignant lesion, 4—suspicious of malignant lesion, and 5—malignant lesion for any slice of a conventional CT image alone, which were selected by a radiologist (with 24 years of experience) 1 month before the first session (Table 1). In the second session, one month after the first session, two independent raters recorded reliability scores for a conventional CT image and the water-HAP image obtained from DECT in the same position as the previous one.

### 2.4. Statistical Analysis

Statistical analysis was performed using software (EZR version 1.40, Jichi Medical University Saitama Medical Center) [11]. The sensitivity, specificity, positive predictive values, and negative predictive values for conventional CT images and conventional CT images plus water-HAP images were calculated based on true positive and true negative findings, as described in the same anatomical region. True bone metastases were confirmed with non-contrast CT, bone scintigraphy, and cancer progression. All patients with a score of 2 or higher were considered to have bone metastases, because the highest sensitivity and specificity were achieved using a cut-off value of 2. A score of 1 was considered to have no bone metastases. The intra-observer area under the curve (AUC) for detecting bone metastasis was compared using the receiver operating characteristic analysis. Model performance was also assessed by calculating AUC, as well as improving the predictive accuracy by calculating the integrated discriminant improvement (IDI) and net reclassification improvement (NRI). A weighted Kappa coefficient was used to calculate the inter-observer agreement. *p*-Values <0.05 indicated statistical significance. Post-hoc power analysis was retrospectively performed using a free software (G* power version 3.1.9.5, Heinrich Heine University, Duesseldorf, Germany; Available at: www.psychologie.hhu.de/arbeitsgruppen/allgemeine-psychologie-und-arbeitspsychologie/gpower.htm) for each rater’s confidence score in patients with true bone metastases.

## 3. Results

**Figure 1:**
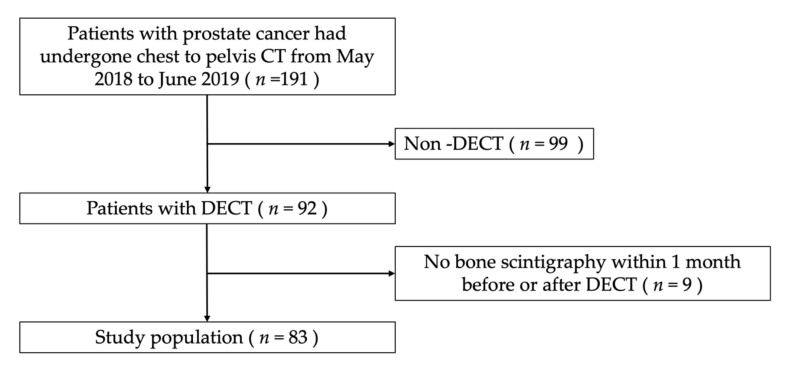
Study flow chart. DECT, Dual-energy computed tomography; PC, Prostate cancer; BC, Breast cancer; HAP Hydroxyapatite.

The final study population was 83 patients (mean age of 76.1 ± 8.7 years, range; from 48 to 103), comprised of 83 men with prostate cancer (Figure 1).

Table 2 summarises patient characteristics. True bone metastasis was confirmed in 31 (37.3 %) out of the 83 patients.

Table 3 shows the absolute number of suspicious bone metastasis lesions in conventional CT images (session-1) and conventional CT images plus water-HAP images (session-2). The sensitivity for the detection of bone metastases was improved.

Table 4 shows the sensitivity, specificity, and negative and positive predictive value of patients with conventional CT images (session-1) and conventional CT images plus water-HAP images (session-2). In session-1, the AUC was 0.861 for rater 1 and 0.845 for rater 2 on the conventional CT images. In session-2, the AUC was 0.990 (*p*-value < 0.01) for rater 1 and 1.000 (*p*-value < 0.01) for rater 2 on the conventional CT images plus water-HAP images.

**Figure 2:**
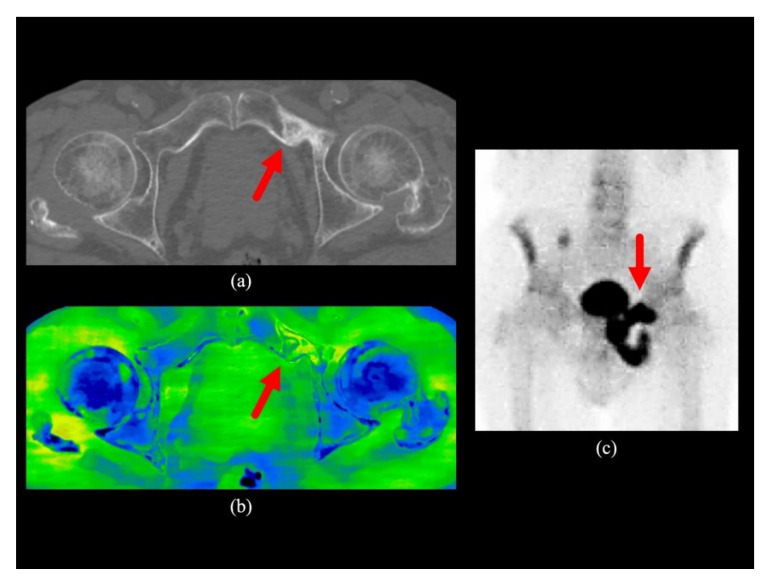
Clinical case 1. A man in his 80s with bone metastasis of prostate cancer. (**a**) The transaxial image of the conventional CT of the pelvis shows the sclerotic bone metastasis in the left superior pubic branch; (**b**) The transaxial water-HAP image of the pelvis shows a localised increase in water density compared to the other side (red arrow); (**c**) The anterior view of the bone scintigraphy shows increased uptake in the same region (red arrow). HAP; Hydroxyapatite.

**Figure 3:**
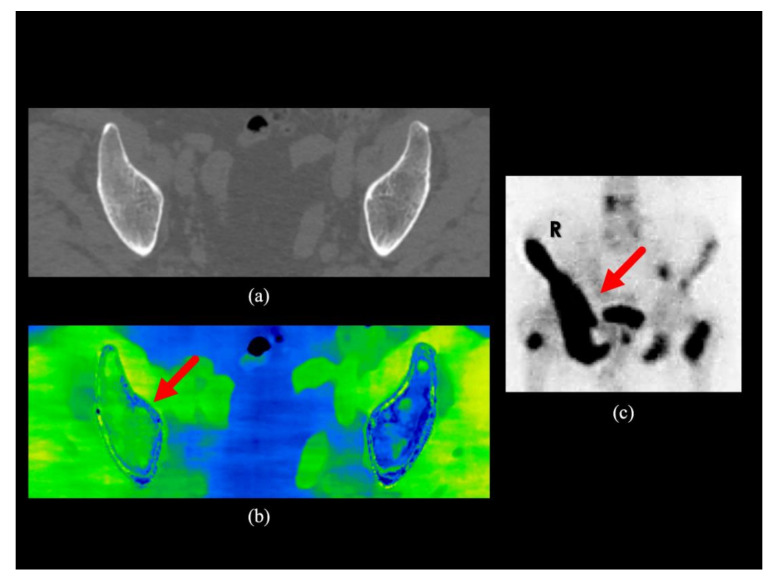
Clinical case 2. A man in his 70s with prostate cancer. (**a**) The transaxial image of the conventional CT of the pelvis does not indicate abnormal concentrations in the iliac bone; (**b**) There is an increase in water density compared to the contralateral side in the transaxial water-HAP image of the pelvis (red arrow); (**c**) The anterior view of the bone scintigraphy shows the right iliac bone metastases (red arrow). HAP; Hydroxyapatite.

**Figure 4:**
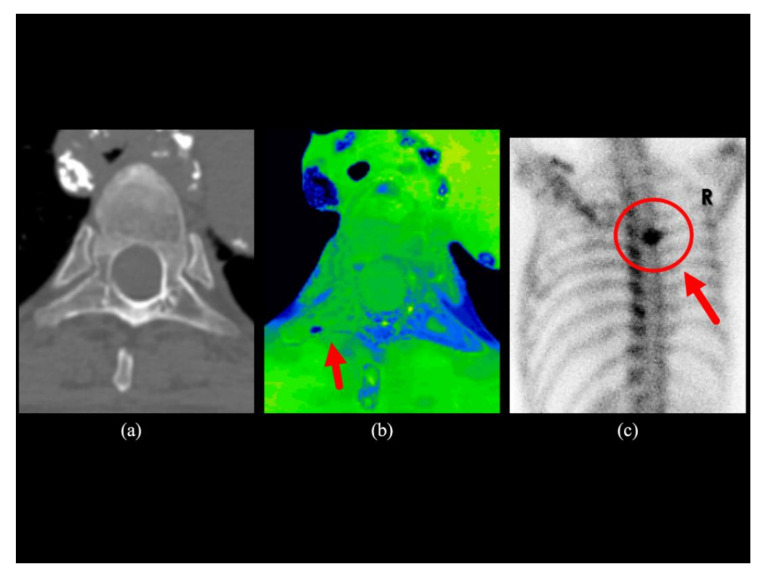
Clinical case 3. A man in his 70s with prostate cancer. (**a**) The transaxial image of the conventional CT image does not indicate an abnormal concentration in the fifth thoracic vertebra; (**b**) There is an increase in water density compared to the contralateral side in the right rib process of the 5th thoracic vertebra in the transaxial water-HAP image; (**c**) The posterior view of bone scintigraphy of shows metastasis to the right posterior element of the fifth thoracic vertebra. HAP; Hydroxyapatite.

The inter-observer weighted kappa coefficient was 0.964 for conventional CT images and 0.976 for conventional CT images plus water-HAP images. The power was calculated as 0.99 for rater 1 and 0.99 for rater 2 using post-hoc power analysis. The parameters used for the post-hoc power test are: test family, t-tests; statistical test, Means: Difference between two dependent means (matched pairs); Type of power analysis, Post-hoc: Compute achieved power; Tails, Two; Effect size d, 0.56 for rater 1, 0.57 for rater 2; α error, 0.05. Representative cases are shown in Figure 2, Figure 3 and Figure 4.

## 4. Discussion

**Figure 5:**
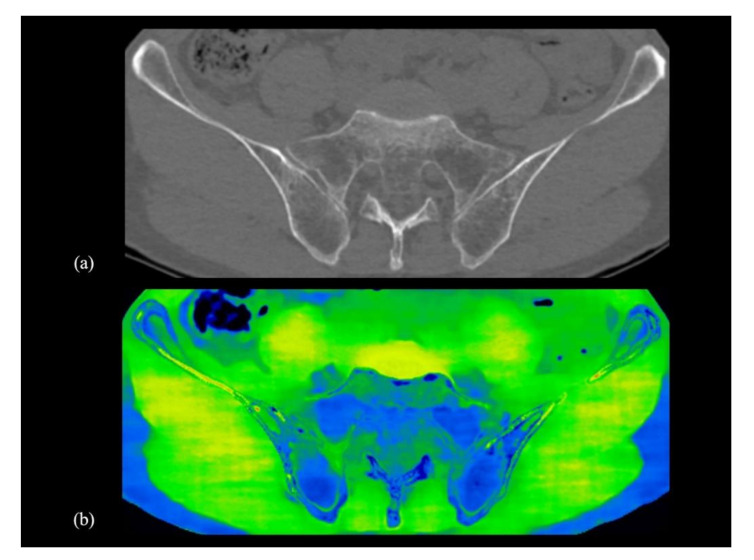
Axial view of the pelvic bones of conventional CT image and water-HAP image in an oncologic patient without bone metastasis in his 70s. (**a**,**b**) The pelvic bone’s water density is comparable to that of subcutaneous adipose tissue and lower symmetrically than that of skeletal muscle. This finding suggests the absence of bone tumours. HAP; Hydroxyapatite.

Water-HAP images combined with conventional CT images yielded increased ability to detect bone metastases, since the ability to detect bone marrow metastases that do not show abnormal density on conventional CT images was significantly increased (an increase of about 10 % in patients with suspected bone metastases). The addition of water-HAP images to plain CT was suggested to improve the diagnostic accuracy of initial staging. In subjects without bone metastases, bone water density was uniformly comparable to that of subcutaneous adipose tissue, which was symmetrically lower than that of skeletal muscle. (Figure 5). There was a heterogeneous increase in water density in sclerotic and mixed-type bone metastases compared to normal bones (Figure 2). In contrast, the water density showed a relatively homogeneous increase in bone marrow metastases (Figure 3 and Figure 4).

The utility of DECT in musculoskeletal imaging has been reported [12,13,14,15,16,17,18,19,20,21,22,23]. Although there are various applications of DECT, the detection of bone marrow oedema and bone contusion has recently been reported in the context of water-HAP images [7,24]. Water-HAP image is a reconstruction technique for visualising water density, but its reports are limited. In the past, it has been reported that the water-HAP image was useful in detecting hip bone marrow oedema in non-traumatic hip pain [7]. The potential of water-HAP using a phantom simulating metastatic bone tumour and small clinical cases has also been reported [8], and these observations support our study findings. Traumatic bone lesions may show a higher degree of oedema and inflammation (water density) than metastatic bone lesions. However, our results showed that the recent DE technique, i.e., water-HAP images, can detect the increased water density in the bone lesions. The degree of increase in water density can potentially characterise the bone lesions, although further studies are warranted.

MRI clearly and accurately depicts the bone metastasis with good contrast [9], but the whole-body screening is difficult in most institutions. In recent years, there have been reports of screening for bone metastases using whole-body diffusion-weighted image (DWI), and whole-body screening is possible in a short time, but the detection sensitivity for bone marrow metastasis is said to be about 70%. However, if a T1-weighted image or short T1 inversion recovery (STIR) image is also added, the detection sensitivity is sufficient, but the imaging time becomes much longer instead, which is not suitable in clinical practice [25,26]. In MRI, one of the solutions is to improve the throughput by further reducing the imaging time using deep learning reconstruction [27]. Still, the problem of claustrophobia remains unsolved, and the DECT detection of bone metastasis may also be helpful in such patients. Nuclear medicine imaging such as 18F-FDG PET/CT and 18F-NaF PET/CT is also a useful modality to detect bone metastases [2,5,28,29,30]. However, it is generally expensive, limited access, and is time-consuming, and it is not performed in many cancer patients.

In contrast, CT may be performed in almost all cancer patients to evaluate the primary and metastatic lesions. Therefore, we believe our results of the added value of water-HAP imaging in assessing bone metastasis can help accurate cancer staging without additional cost and reduce radiation exposure. In contrast, the inter-observer agreement score did not change significantly after the addition of water-HAP images. Its addition also did not increase the inter-observer agreement in the present study.

This study had some limitations. First, it was a single-centre retrospective study. Secondly, the only tumour studied is prostate cancer; thereby, the results cannot be generalised to other malignancies. However, the sensitivity of conventional CT images is about 70%, and the specificity is about 98%, which are in line with the previous reports [31,32]. In conventional CT images plus water-HAP images, both sensitivity and specificity have very high values. Specifically, the sensitivity of both raters 1 and 2 was 1.000. Meanwhile, their specificities were 0.981 and 1.000, respectively), which might be the effect of selecting an arbitrary slice. Third, there was no pathological confirmation of bone metastases. However, clinically, the diagnosis of bone metastases from prostate cancer was clear.

We used the DECT technique to detect bone metastases by tissue characterisation. There were two advantages in this technique, i.e., no requirement of a contrast agent and no increase in radiation exposure. The DE system of this device does not change or increase the amount of radiation exposure compared to the conventional device [33], and the increase in the ability to detect bone metastases due to the addition of new technology to widely used CT greatly contributes to the increase in the accuracy of the initial staging of cancer. In this regard, the use of DECT in clinical settings can be positively recommended. Another potential approach to identifying the bone metastasis may be detecting “iodine density” in the lesion on contrast-enhanced CT, but it requires the contrast agent. Further comparison study should be necessary, and it is now underway in our laboratory.

## 5. Conclusions

In conclusion, water-HAP images not only enhance the ability to detect bone metastases from prostate cancer but also optimises patient management. Specifically, CT with water-HAP images reduces the need for additional radiographic imaging, potentially reducing costs and radiation exposure. Further larger population studies are needed, such as quantitative analysis, various cancer metastases, and comparison with MRI images and histopathological examination.

## Figures and Tables

**Table 1:** Visual evaluation score for bone lesions in conventional CT images and conventional CT images plus water-HAP images.

Score	Conventional CT Images	Conventional CT Images Plus Water-HAP Image
1	No abnormality or findings of apparent benign bone lesions on conventional CT images	No abnormality on conventional CT images and water-HAP images, and apparent benign bone lesions on conventional CT images
2	Do not qualify as score 1, 3, 4, or 5	Do not qualify as score 1, 3, 4, or 5 on conventional CT images. Water-HAP images show a focal asymmetric density lower than skeletal muscle but higher than surrounding bone marrow, but conventional CT images do not indicate any abnormalities
3	Ill-defined solitary sclerotic or lytic lesion	Ill-defined solitary sclerosis or lytic lesion on conventional CT images, or water-HAP images show focal asymmetrical density equal to or higher than skeletal muscle, but no abnormal density was found on conventional CT images
4	Ill-defined multiple sclerotic or lytic lesions	Ill-defined multiple sclerosis or lytic lesions, or water-HAP images show focal asymmetrical density equal to or higher than skeletal muscle consistent with an ill-defined bone sclerotic or lytic lesion on conventional CT images
5	Apparent malignant bone lesions with bone destruction or extraosseous mass on conventional CT images	Apparent malignant lytic lesion or bone sclerosis with bone destruction or extraosseous mass on conventional CT images

HAP—hydroxyapatite.

**Table 2:** Patient characteristics.

Patients Characteristics	Value
Mean age ± S.D. (years)	76.1 ± 8.7
Men (*n*)	83
Bone metastasis (*n*)	
Positive	31
Negative	52

S.D.: standard deviation.

**Table 3:** Number of cases scored on conventional CT images and conventional CT images plus water-HAP.

Score	Conventional CT Images		Conventional CT Image Plus Water-HAP Images	
	rater 1	rater 2	rater 1	rater 2
1 (normal or benign)	59 (71.1 %)	60 (72.3 %)	51 (61.4 %)	52 (62.7 %)
2 (probably benign)	6 (7.2 %)	5 (6.0 %)	3 (3.6 %)	3 (3.6 %)
3 (unclear)	3 (3.6 %)	5 (6.0 %)	10 (12.0 %)	10 (12.0 %)
4 (probably malignant)	5 (6.0 %)	2 (2.4 %)	9 (10.8 %)	6 (7.2 %)
5 (malignant)	10 (12.0 %)	11 (13.3 %)	10 (12.0 %)	12 (14.5 %)
Total	83 (100 %)	83 (100 %)	83 (100 %)	83 (100 %)

HAP—hydroxyapatite.

**Table 4:** Diagnostic performance of conventional CT images and conventional CT images plus water-HAP images.

		Rater 1			Rater 2		
Session	Parameter	Value	95% CI	*p*-Value	Value	95% CI	*p*-Value
Conventional CT images	Sensitivity	0.742	0.643–0.768		0.710	0.610–0.736	
	Specificity	0.981	0.922–0.997		0.981	0.921–0.997	
	PPV	0.958	0.830–0.992		0.957	0.822–0.992	
	NPV	0.864	0.812–0.878		0.850	0.798–0.864	
	AUC	0.861	0.781–0.942	<0.01	0.845	0.762–0.929	<0.01
Conventional CT images plus water-HAP images	Sensitivity	1.000	0.931–1.000		1.000	0.941–1.000	
	Specificity	0.981	0.940–0.981		1.000	0.965–1.000	
	PPV	0.969	0.902–0.969		1.000	0.941–1.000	
	NPV	1.000	0.958–1.000		1.000	0.965–1.000	
	AUC	0.990	0.972–1.000	<0.01	1.000	1.000–1.000	<0.01
	continuous NRI	0.867	0.614–1.119	<0.01	0.867	0.614–1.119	<0.01
	IDI	0.742	0.376–1.108	<0.01	0.806	0.423–1.190	<0.01

HAP—hydroxyapatite, CI—confidence interval, PPV—positive predictive value, NPV—negative predictive value, AUC—area under the curve, NRI—net classification improvement, IDI—integrated discrimination improvement.

## Abbreviations

AUC	Area under the curve
CI	Confidence interval
CT	Computed tomography
DECT	Dual-energy computed tomography
HAP	Hydroxyapatite
MRI	Magnetic resonance imaging
NPV	Negative predictive value
PPV	Positive predictive value
ROC	Receiver operating characteristic
